# Shifting Information-Seeking Behaviors of Rural Older Americans During the Era of the COVID-19 Pandemic

**DOI:** 10.1093/geroni/igab046.2671

**Published:** 2021-12-17

**Authors:** Brady Lund

**Affiliations:** Emporia State University, Wichita, Kansas, United States

## Abstract

This session reports findings on how older rural adults in the Midwest United States adapted their information seeking behaviors in the face of the COVID-19 pandemic. A series of nearly three-dozen interviews conducted during late-summer 2020 capture the experiences of members of this population in their own words. Findings indicate that the experiences of the rural older American during this period were often unique to each individual and cannot be easily explained by a single social or demographic factor. Those participants with greater educational attainment were more likely to use a variety of digital technologies (smartphones, tablets, at-home personal computers) prior to the pandemic and thus experienced fewer challenges finding reliable information when the pandemic began. Those who were married felt less socially-isolated, but were often more reliant on others to find information for them. Women were more likely than people with other gender identities to use social media to connect and find information, which helped abate feelings of isolation but also made them feel more susceptible to misinformation and information overload. All participants expressed some level of fear or concern that motivated them to seek health information, while many expressed the same motivation in seeking political and economic information. These findings suggest that the information seeking behaviors of rural older adults were dramatically altered by the COVID-19 pandemic, with most becoming more reliant on digital technology to find information, and all being motivated by fear, concern, and/or curiosity to find information about the pandemic.

